# Dynamic functional connectivity estimation for neurofeedback emotion regulation paradigm with simultaneous EEG-fMRI analysis

**DOI:** 10.3389/fnhum.2022.933538

**Published:** 2022-09-16

**Authors:** Raziyeh Mosayebi, Amin Dehghani, Gholam-Ali Hossein-Zadeh

**Affiliations:** ^1^School of Electrical and Computer Engineering, University of Tehran, Tehran, Iran; ^2^School of Cognitive Sciences, Institute for Research in Fundamental Sciences (IPM), Tehran, Iran

**Keywords:** neurofeedback, dynamic connectivity, EEG, fMRI, Correlated Coupled Tensor Matrix Factorization (CCMTF)

## Abstract

Joint Analysis of EEG and fMRI datasets can bring new insight into brain mechanisms. In this paper, we employed the recently introduced Correlated Coupled Tensor Matrix Factorization (CCMTF) method for analysis of the emotion regulation paradigm based on EEG frontal asymmetry neurofeedback in the alpha frequency band with simultaneous fMRI. CCMTF method assumes that the co-variations of the common dimension (temporal dimension) between EEG and fMRI are correlated and not necessarily identical. The results of the CCMTF method suggested that EEG and fMRI had similar covariations during the transition of brain activities from resting states to task (view and upregulation) states and these covariations followed an increasing trend. The fMRI shared spatial component showed activations in the limbic system, DLPFC, OFC, and VLPC regions, which were consistent with the previous studies and were linked to EEG frequency patterns in the range of 1–15 Hz with a correlation value close to 0.75. The estimated regions from the CCMTF method were then used as the candidate nodes for dynamic functional connectivity (dFC) analysis, in which the changes in connectivity from view to upregulation states were examined. The results of the dFC analysis were compared with a Normalized Mutual information (NMI) based approach in two different frequency ranges (1–15 and 15–40 Hz) as the NMI method was applied to the vectors of dFC nodes of EEG and fMRI data. The results of the two methods illustrated that the relation between EEG and fMRI datasets was mostly in the frequency range of 1–15 Hz. These relations were both in the brain activations and the dFCs between the two modalities. This paper suggests that the CCMTF method is a capable approach for extracting the shared information between EEG and fMRI data and can reveal new information about brain functions and their connectivity without solving the EEG inverse problem or analyzing different frequency bands.

## Introduction

Neurofeedback is a non-invasive brain training technique for modulation of brain activity/function with several medical and non-medical applications like emotion regulation (Hammond, 2005; Sterman and Egner, 2006; Jarusiewicz, 2008; Johnston et al., 2010; Scharnowski et al., 2012; Enriquez-Geppert et al., 2019; Van Doren et al., 2019). In neurofeedback as self-regulation of brain activity/function, the brain activity/function is acquired, quantified, and then returned to the participant to modulate and regulate the brain mechanism toward a special level or direction. EEG and fMRI are two neuroimaging modalities used for emotion regulation neurofeedback. EEG frontal asymmetry was used in several previous studies for emotion regulation using the power spectrum of frontal EEG channels in the special frequency band (Peeters et al., 2014; Allen and Reznik, 2015; Quaedflieg et al., 2015; Kelley et al., 2017; Mennella et al., 2017; Reznik and Allen, 2018; Dehghani et al., 2020b). For emotion regulation based on fMRI neurofeedback, amygdala activity as feedback was used in several previous neurofeedback studies (Zotev et al., 2016; Barreiros et al., 2019).

EEG and fMRI modalities record different aspects of brain activations. While EEG acquires neural oscillations, fMRI records the Blood Oxygen Level Dependent (BOLD) signal which reflects changes in deoxyhemoglobin and blood oxygenation coupled to underlying neuronal activity. EEG has high temporal resolution while its spatial resolution is low. On the other hand, fMRI has a high spatial resolution, but its temporal resolution is low and is affected by Hemodynamic Response Function (HRF). Therefore, the two modalities can be used simultaneously to compensate for the deficiencies of one another (Cichy and Oliva, 2020). Recent advances in data acquisition have made it possible to simultaneous recording of these two modalities and it utilizes the advantages of both modalities. The effectiveness of simultaneous EEG and fMRI neurofeedback was demonstrated in previous studies (Zotev et al., 2014, 2018b; Zich et al., 2015; Perronnet et al., 2017; Lioi et al., 2018, 2020). Integration of EEG and fMRI provides a better understanding of brain mechanisms during neurofeedback, especially because of the low spatial or temporal resolution that each modality has it alone. Therefore, the fusion of EEG and fMRI modalities provides complementary and valuable information for a deep understanding of brain mechanisms during neurofeedback and may help to explain and extract the Spatio-temporal brain regions which are undetectable with only one modality (Daunizeau et al., 2007).

In recent years, several methods have been developed for the fusion of EEG and fMRI. Among them, several methods are based on joint factorization of these two modalities such as Canonical Correlation Analysis (Correa et al., 2010), joint Independent Component Analysis (ICA) (Moosmann et al., 2008), and Independent Vector Analysis (Lee et al., 2008) in which a matrix representation of data is employed and an objective function is minimized to find out the common profiles. On the other side, there is N-way Partial Least Square (N-PLS) (Martínez-Montes et al., 2004), Advanced Coupled Matrix Tensor Factorization (ACMTF) (Acar et al., 2014), and Correlated Coupled Matrix Tensor Factorization (CCMTF) (Mosayebi and Hossein-Zadeh, 2020) methods based on matrix and tensor representation of the two modalities in which an objective function is defined such that the common signature between a matrix and a tensor is estimated.

Tensors are arrays with higher dimensions. Therefore, for multi-dimensional data such as EEG (channel, Spectrum, trial, and so on), tensors are proper choices and permit the natural representation of the dataset. Also, with tensor-based methods for fusion of EEG and fMRI data, the relation of more features is extracted between the two modalities. For example, the relation between the EEG spectrum and channel maps with the fMRI spatial maps are extracted while an amplitude modulation of these signatures is also estimated. In this paper, we used the CCMTF method to estimate the shared and hidden information between EEG and fMRI datasets through neurofeedback and retrieve the positive autobiographical paradigm. Then, the extracted fMRI spatial information is used for state-wise connectivity analysis to analyze the connectivity changes across the transition of different states.

CCMTF method was introduced by Mosayebi and Hossein-Zadeh (2020). This method has superiority over the other two tensor-based methods (ACMTF and N-PLS). The ACMTF method considers an identical shared profile between the two datasets, which is a confining assumption and is not always acceptable in two different modalities such as EEG and fMRI with many differences in their physiological measures. On the other hand, compared to the N-PLS method, in the CCMTF method, the significance of each component is estimated, and the interpretation of the results is more justifiable in the case of over-factorization. In Mosayebi and Hossein-Zadeh (2020), the authors have made a full comparison between these methods. As a result, employing the CCMTF method for the fusion of EEG and fMRI datasets has more advantages compared with the other methods and makes it a proper choice for the fusion analysis of EEG and fMRI data, especially in an emotion neurofeedback paradigm for a deep understanding of brain mechanisms.

The details of EEG and fMRI datasets used in this study were described in Dehghani et al. (2020a). The task contained three main blocks, named “Rest,” “View,” and “Upregulate.” These datasets are arranged such that the common profile is considered across the state mode and reflects the amplitude modulation of other features, such as EEG Spectrum, EEG channel maps, and fMRI spatial profile. We estimate the fMRI spatial locations that have similar amplitude modulation in EEG data. The estimated brain regions are then compared with the results of the GLM analysis and ICA method. The corresponding frequency content and channel map are also estimated simultaneously in the CCMTF method.

The candidate regions are then employed as the nodes of a connectivity graph to analyze the variation of connections between these nodes during the transition from view to upregulation states. In the sham group, the connectivity graph is also analyzed in the candidate regions to determine the effect of the neurofeedback on the connectivity among the selected nodes. To form the connectivity graphs, Pearson correlation is used between the BOLD signals in each Region of Interest (ROI). The results of the connectivity analysis are then compared with a previous study (Wirsich et al., 2020). In this study, a Normalized Mutual Information (NMI) method is used to extract the shared dynamics in the matrix of EEG and fMRI connectivity.

This paper is organized as follows. In section “Materials and methods,” the tensor factorization, the CCMTF method, and the NMI approach are briefly introduced. The experimental paradigm and application of the CCMTF method on the experimental data are described in section “Data.” The results are illustrated in section “Results,” and sections “Discussion” and “Conclusion” are devoted to discussion and conclusion of the paper.

## Materials and methods

### Tensor decomposition

Tensors are multiway arrays with higher dimensions. Principle Component Analysis, Singular Value Decomposition, or other matrix factorization methods employ two features of multi-dimensional datasets. For multivariate data such as EEG, tensors are useful for the estimation of the interdependency between different features and the underlying profiles. A multiway tensor χ can be decomposed using different techniques. Among these techniques, Canonical Polyadic Decomposition (CPD) or PARAFAC is very noticeable because of its unique property. Besides CPD, Block Term Decomposition and Tucker decomposition are other widely used methods. Consider the χ ∈ ℝ*^I^*^×*M*×^*^N^* as a three-way tensor, then its CP decomposition is written as follows (Yilmaz et al., 2011):


(1) $$\chi =\sum_{r=1}^{R}\lambda_{r}\,a_{r}^{{}^{\circ}}\,b_{r}^{{}^{\circ}}\,c_{r}$$

where, *a_r_*, *b_r_*, and *c_r_* are the components in the first, second and third mode, respectively, *R* is the rank of χ and λ_*r*_ is the corresponding weight. The symbol “*o*″ represents the vector outer product. We use this decomposition in the next section.

### Correlated coupled matrix tensor factorization

CCMTF method is developed for the fusion of a tensor and a matrix with similar covariations in their common profiles. The objective function is expressed as follows:


(2) $$\begin{array}{l} g(A_{y},B,A_{x},C,D)\;={\left\|Y-A_{y}\Sigma B^{T}\right\|}^{2}\\ \;\;\;\;\;\;\;\;\;\;\;+{\left\|\chi -[[\lambda ;A_{x},C,D]]\right\|}^{2}\\ \;\;\;\;\;\;\;\;\;\;\;+\mu \sum_{r=1}^{R}\left(1-e^{-\frac{{\left(\sigma_{r}\lambda_{r}\right)}^{2}}{\varepsilon }}\right)\\ \;\;\;\;\;\;\;\;\;\;\;\left(1-C\left(a_{yr},a_{xr}\right)\right)\\ \;\;\;\;\;\;\;\;\;\;\;+\beta {\left\|\lambda_{r}\right\|}_{1}+\beta {\left\|\sigma_{r}\right\|}_{1}\\ \;\;\;\;\;\;\;\;\;\;\;s.t.\left\|a_{yr}\right\|=\left\|b_{r}\right\|=\left\|a_{xr}\right\|\\ \;\;\;\;\;\;\;\;\;\;\;=\left\|c_{r}\right\|=\left\|d_{r}\right\|=1\\ \;\;\;\;\;\;\;\;\;\;\;\;for\;r=1,\ldots ,R \end{array}$$

*Y* is the data matrix in which *A_y_* and *B* are its two underlying factors in a singular value decomposition approach and Σ is the diagonal matrix with σ as its diagonal entries. χ is the data tensor in which *A_x_*, *C*, and *D* are its three underlying factors in a Canonical Polyadic Decomposition approach. *A_y_* and *A_x_* are the common profiles between the two datasets and λ is the weight (singular value). *T* is denoted as matrix transpose, μ, ε, and β are penalty parameters which are described in Mosayebi and Hossein-Zadeh (2020), *R* is the total rank of datasets and C is defined as the squared correlation between the corresponding components of the common profiles as follow:


(3) $$C\,\left(a_{x\,r},a_{y\,r}\right)={\left(c\,o\,r\,r\,\left(a_{x\,r},a_{y\,r}\right)\right)}^{2}={\left(\frac{{\left(a_{x\,r}-\bar{a_{x\,r}}\right)}^{T}\,\left(a_{y\,r}-\bar{a_{y\,r}}\right)}{||\left(a_{x\,r}-\bar{a_{x\,r}}\right)||\,||\left(a_{y\,r}-\bar{a_{y\,r}}\right)||}\right)}^{2}$$

Components are the columns of each factor. The shared components are discriminated against with unshared components using the values of λ and σ. If these two weights have simultaneously significant values for two corresponding components in datasets, these two components are considered shared, otherwise, the components are unshared. In the third term in (2), $\left(1)e^{-\frac{{\left(\sigma_{r}\lambda_{r}\right)}^{2}}{\varepsilon }}\right)$ is defined as an adaptive penalty parameter in Mosayebi and Hossein-Zadeh (2020), that limits the significance of the third term only to the shared components. Therefore, only the correlation between the shared components is maximized.

### Normalized mutual information

The results of the current study are compared with a method based on NMI, which has been published in Wirsich et al. (2020). NMI is computed for vectors of dynamic functional connectivity (dFC) of two identical nodes between EEG and fMRI datasets. The NMI metric is:


(4) $$Y\,\left(E,F\right)=\frac{H\,\left(E\right)+H\,\left(F\right)}{H\,\left(E,F\right)}$$

In which, *H*(*E*) and *H*(*F*) are the entropies of EEG and fMRI dFCs and *H*(*E*,*F*) is the joint entropies of two dFCs.

## Data

### Neurofeedback paradigm and data acquisition

The research protocol was approved by the ethics committees of the Iran University of Medical Sciences, Tehran, Iran. 18 healthy subjects (age 26.7 3.6 years, all-male as the experimental group, and 14 healthy subjects (age 27 3.8 years, all-male) as the control group participated in this study. The paradigm and data collection were described in detail in previous studies (Dehghani et al., 2020a,b, 2021, 2022). As a short description, the experimental paradigm in this study was according to retrieving positive autobiographical memories and the effectiveness of the neurofeedback paradigm was demonstrated in the previous studies (Dehghani et al., 2020a,b, 2021, 2022). The experimental paradigm included 10 runs of three blocks, namely, rest, view, and upregulation with a duration of 20, 40, and 60 s with 4–6 s intervals between View and Upregulation blocks. During the rest block, participants were asked to relax without doing anything. In the view block, two pictures (from individual positive autobiographical memories) were presented for 40 s and participants were asked to see them without remember anything. In the upregulation block, two images like the previous view block were presented and participants were asked to retrieve the autobiographical positive memories related to the presented images and increase the height of the neurofeedback bar. Neurofeedback was presented only in the upregulation period based on the approach–withdrawal hypothesis (Davidson, 1998) and was calculated as the difference between the EEG power in the right and left hemispheres in the alpha frequency band in 2 s time windows, updated every 1 s with 50% overlap between the consecutive windows. Participants in the control group received sham neurofeedback according to the random signal proposed in Dehghani et al. (2020a,b).

The MRI data were acquired using a 3 Tesla Scanner (Prisma, Siemens, Erlangen, Germany) located in the National Brain Mapping Lab (NBML), Tehran, Iran. Functional MRI were acquired using a T2*-weighted gradient-echo, echo-planar (EPI) pulse sequence (TR = 2,000 ms, TE = 30 ms, flip angle = 90°, matrix size = 64 × 64 × 30, and voxel size = 3.8 × 3.8 × 4 mm). Structural images were acquired using a gradient-echo, T1-weighted MPRAGE pulse sequence (TI = 1,100 ms, TR = 1,810 ms, TE = 3.47 ms, and voxel size = 1 × 1 × 1 mm).

The 64-channel EEG data were recorded at 5K samples/s simultaneously with fMRI using an MRI-compatible EEG system (Brain Products, München, Germany) according to the 10–20 system.

### Correlated coupled tensor matrix factorization for neurofeedback paradigm

Before applying the CCMTF method, it is necessary to choose the common profile between EEG and fMRI data. Preprocessing pipline of EEG and fMRI were completely described in previous study (Dehghani et al., 2020b). For EEG data, several steps, e.g., removing fMRI artifact, detecting the QRS complexes from an ECG channel, removing the pulse (ballistocardiography/BCG), and finally ICA for removing eye blinking, head movement, and cardioballistic or BCG residual were done. For fMRI data, preprocessing methods like slice-timing correction, motion correction, temporal high pass filtering (cut-off = 0.005 Hz), and spatially smoothing using an 8 mm full-width at half-maximum Gaussian kernel were applied. Considering the paradigm for data acquisition, in each run, we have three distinctive states. At first, the resting state with a total duration of 20 s is recorded, followed by a 40 s duration of view state, in which two different pictures are shown to the subject, each with a duration of 20 s. Then, the upregulation state is recorded which has a total duration of 60 s, in which two different pictures are shown alongside the neurofeedback signal and each picture has a duration of 30 s. In each run, we have considered four different features for analyzing the data, including states, spectrum, and channel maps for EEG and states and spatial locations for fMRI. The state feature is the common profile between the two modalities. In the other words, in each state, we computed the spectrum of EEG signals for each electrode and then arrange the resulting data in a matrix. This procedure is repeated for the next runs. We divided the view and upregulation states into two similar states corresponding to each illustrated picture, to have more data in the state mode. Therefore, in state mode, there are 10 rows corresponding to the rest state, 20 rows corresponding to the view state, and 20 rows corresponding to upregulation state, resulting in 50 rows in state mode. In each state row, we have a matrix computed from EEG data in the spectrum and channel dimensions. For fMRI data, the BOLD signal is averaged during the data acquisition procedure in each state. As a result, for each state of fMRI data, we have a vector corresponding to the average BOLD value of each voxel of the brain. The idea of averaging the BOLD signal stems from the fact that the average BOLD signal is increased during the neurofeedback in comparison to the other two states (Birbaumer et al., 2009; Dehghani et al., 2020b).

As a result, we have a three-way array for EEG data with state, spectrum, and channel topoplots as its three modes and a matrix for fMRI data with state and voxels as its two modes. The dimension of state mode, spectrum mode, channel mode, and voxels mode are 50, 256, 64, and 63,665, respectively.

The CCMTF method is then applied to the configured data sets as follows:


(5) $$\begin{array}{l} g\left(S_{f},V,S_{e},F,C\right)\;={\left\|Y-S_{f}\Sigma V^{T}\right\|}^{2}\\ \;\;\;\;\;\;\;\;\;\;\;+{\left\|\chi -[[\lambda ;S_{e},F,M]]\right\|}^{2}\\ \;\;\;\;\;\;\;\;\;\;\;+\mu \sum_{r=1}^{R}\left(1-e^{-\frac{{\left(\sigma_{r}\lambda_{r}\right)}^{2}}{\varepsilon }}\right)\\ \;\;\;\;\;\;\;\;\;\;\;\left(1-C\left(s_{fr},s_{er}\right)\right)\\ \;\;\;\;\;\;\;\;\;\;\;+\beta {\left\|\lambda_{r}\right\|}_{1}+\beta {\left\|\sigma_{r}\right\|}_{1}\\ \;\;\;\;\;\;\;\;\;\;\;s.t.\left\|s_{fr}\right\|=\left\|v_{r}\right\|=\left\|s_{er}\right\|\\ \;\;\;\;\;\;\;\;\;\;\;=\left\|f_{r}\right\|=\left\|m_{r}\right\|=1\\ \;\;\;\;\;\;\;\;\;\;\;for\;r=1,\ldots ,R \end{array}$$

Where, *Y* and χ represent the fMRI and EEG datasets, respectively. *S_f_* and *S_e_* are the state factor of fMRI and EEG data, *V* is voxel factor, *F* is frequency spectrum factor, and *M* is channel map factor. The objective function in (5) factorizes EEG tensor and fMRI matrix such that the shared components of the common profile (state mode) are maximally correlated. Therefore, we find the underlying amplitude modulation across the states captured by both EEG and fMRI datasets.

**FIGURE 1:**
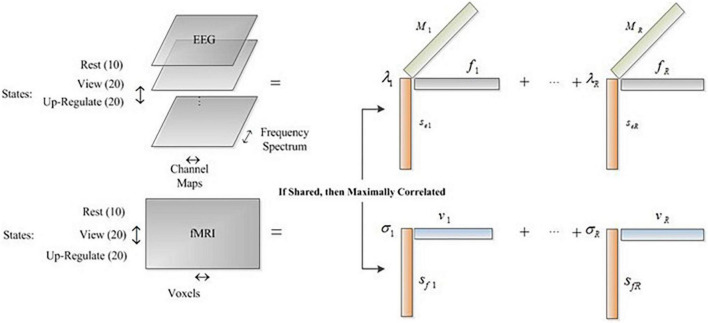
The arrangement of datasets for CCMTF method.

As it is shown in Figure 1, the common mode is the state feature which depicts the variation of the other features (EEG spectrum and channel map and fMRI spatial component) over time. Hereinafter, we call this mode the Amplitude Modulation (AM) mode because it shows the amplitude modulation of the other features in the datasets. It is noteworthy to mention that we extract the common neural activities between EEG and fMRI which their amplitude modulation profiles in these three states are maximally correlated.

## Results

### Results of the correlated coupled tensor matrix factorization method

The CCMTF method is applied to the configured datasets. One important point before applying the CCMTF method is to select the rank of the dataset. For this purpose, the rank of the EEG tensor is computed using the Corcondia test. The best rank according to the value of the Corcondia test is 2. To choose the rank of the total dataset, we start from this value and compute the components for larger values of *R*. As we increase *R*, we observe that repetitive components or some components with no biological concept are estimated. Therefore, the value of *R* is selected as equal to 2. Besides, for *R* greater than 2, some of the weights in EEG and fMRI data become simultaneously zero, which indicates the over-factorization problem. The most important point is that for all values of *R*, only one shared component between EEG and fMRI is extracted and we are interested in this shared component.

**FIGURE 2:**
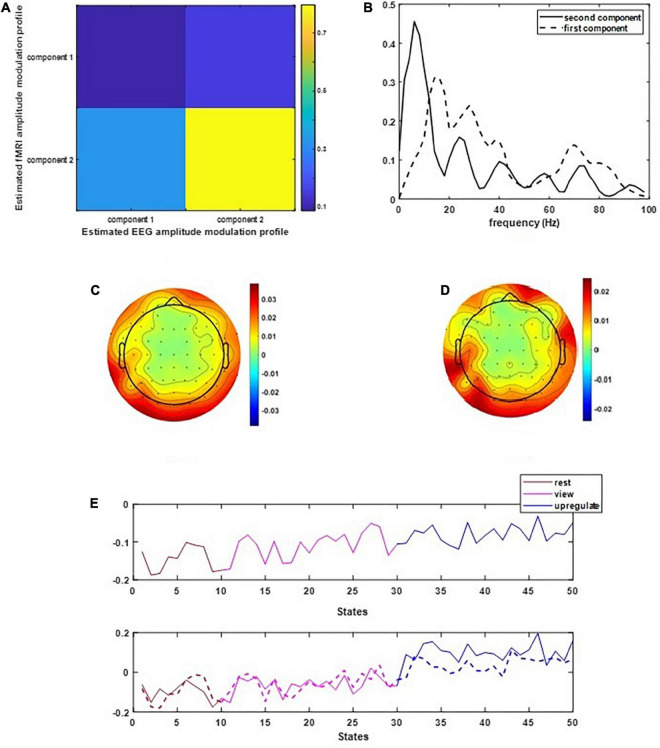
The results of CCMTF method: **(A)** the correlation matrix between EEG and fMRI modulation profiles, **(B)** EEG frequency components, **(C,D)** EEG topoplots, and **(E)** EEG and fMRI amplitude modulation, solid lines show the EEG AM profile.

Figure 2 illustrates the results of applying the CCMTF method. Figure 2A shows the correlation value between the EEG and fMRI components. As can be seen in this figure, the second component of EEG data is correlated with the fMRI component with a correlation value close to 0.75. The first EEG component has no corresponding counterpart in fMRI data and therefore no significant correlation is estimated. Figure 2B shows the frequency spectrum of two EEG components. The unshared component contains a wide range of frequency rhythms from alpha to gamma oscillations. Its corresponding EEG topoplots in Figure 2C show activations in parietal and occipital regions. On the other hand, the shared frequency component contains parts of theta and the whole range of alpha oscillations, in which the EEG topoplots in Figure 2D show neural activation in parietal, occipital and frontal regions (Zotev et al., 2018a).

**FIGURE 3:**
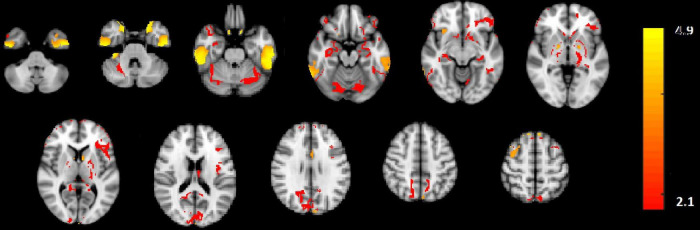
The activated regions in fMRI spatial component that are FDR corrected at the level of 0.05 for multiple comparisons. The slice numbers from left to right are 15, 19, 23, 27, 31, 35, 39, 46, 50, 52, and 56 in MNI coordinate.

In Figure 2E, the amplitude modulation of neural activities across each state follows an increasing trend. This phenomenon shows that the extracted brain activities increase from rest to view and then from view to upregulation (The samples from 1 to 10, 11 to 30, and 31 to 50 are corresponding to resting, view, and upregulation state). In the lower plot of Figure 2E, an interesting jump in EEG component from samples 30 to 31 shows an effective increase in brain activity in upregulation, with respect to the view state. The corresponding fMRI component in Figure 3 illustrates activation in amygdala, caudate, cerebellum, cingulate cortex, cuneus, inferior temporal lobe, insula, hippocampus, palladium, putamen, superior frontal gyrus, superior parietal gyrus, thalamus, middle temporal gyrus, frontal operculum, occipital lobe, middle frontal gyrus, inferior frontal gyrus, and ventral striatum, and corrected for multiple comparisons using false discovery rate (FDR) at the level of 0.05. This change is confirmed by previous results of emotion regulation paradigms (Zotev et al., 2014, 2016; Dehghani et al., 2020a,b), that the neural activation in these regions increases in response to neurofeedback signals. The phenomenon shows the increase of average BOLD signal from rest to view and view to upregulation, which confirms the shape of the estimated AM components in the two modalities.

We also used the results GLM and ICA methods to the fMRI data (Dehghani et al., 2020a,b) for comparison with the CCMTF method. The stimulus pattern is used as the regressor for GLM analysis, and 45 independent components are extracted for the ICA method. The activated regions are reported in Table 1. The extracted brain regions by CCMTF are in limbic, frontal, temporal, and occipital regions. The involvement of these regions justifies according to emotion regulation models in previous studies (Kohn et al., 2014; Dehghani et al., 2020b). As a short description, limbic and deep brain regions like the amygdala, insula, thalamus, caudate, putamen, and palladium play a key role in emotion regulation/generation, recalling positive and negative memories and they connect with several regions involved in emotion regulation especially prefrontal and frontal regions. Prefrontal/frontal cortex regions are involved in a wide range of activities related to emotion and recalling memories. Activation of parietal regions is related to these regions’ role in attention deployment, especially to positive images presented to recall the individual autobiographical (Andersen, 1997; Svoboda et al., 2006; Ochsner and Gross, 2008; Bracht et al., 2009). As is illustrated in Table 1, the active regions obtained by the CCMTF method are similar to those obtained by ICA and GLM methods. However, there are some differences as a result of joint analysis of EEG and fMRI in the CCMTF method.

**TABLE 1:** The extracted activated ROIs obtained by CCMTF, GLM, and ICA methods.

Method	Activated regions
CCMTF	Amygdala, caudate, cerebellum, cingulate cortex, cuneus, inferior temporal lobe, superior frontal gyrus, superior parietal gyrus, middle temporal gyrus, middle frontal gyrus, inferior frontal gyrus (VLPFC, DLPFC, and OFC), frontal operculum, occipital lobe, ventral striatum, insula, hippocampus, palladium, thalamus, putamen
GLM	Cuneus, fusiform, lingual gyrus, middle occipital, thalamus, hippocampus, amygdala, caudate, putamen, insula, ventral striatum, prefrontal, and frontal cortex (VLPFC, DLPFC, and OFC), inferior parietal gyrus, middle temporal gyrus, precuneus, insula
ICA	Cuneus, Precuneus, Posterior Cingulate Cortex, Dorsolateral Prefrontal Cortex, Amygdala, Caudate, Hippocampus, Insula, Putamen, Thalamus, Right Middle Occipital, Fusiform, Ventral Striatum, Lingual Gyrus, Ventrolateral Prefrontal Cortex, Dorsolateral Prefrontal Cortex, Orbitofrontal Cortex, Middle Temporal Gyrus, Inferior Parietal

### Brain connectivity changes

To estimate the dynamics of brain connectivity from the view to the upregulation state, we used the estimated activated regions of the previous section as the candidate nodes of the brain connectivity graph. Therefore, for this analysis, there are 30 candidate nodes for each connectivity matrix in each state. In each state, the correlation coefficient between the averaged BOLD signals of the voxels of each region is computed between the candidate nodes, and a connectivity graph is generated. This graph shows the strength of connectivity between the activated regions of the previous section in each state. Besides the individual connectivity of each state, we want to observe the changes in connectivity during the transition of the states in the neurofeedback paradigm. To accomplish this, we used the two paired *t*-tests on the view-upregulation connectivity matrices. Then, the results are thresholded at the *p*-value of 0.05 to extract the significant variations of connectivity between each pair.

**FIGURE 4:**
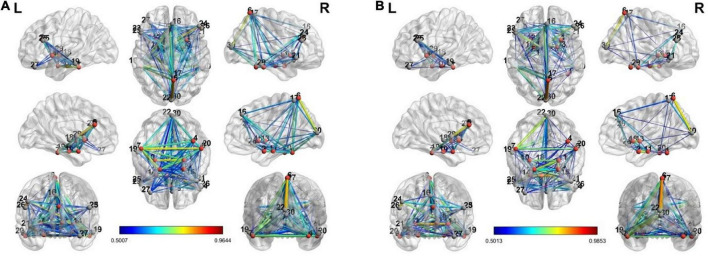
The connectivity graph of view and upregulation states from different views in the brain: **(A)** view state, **(B)** up-regulation state.

The connectivity graph is extracted with the BrainNet Toolbox (Xia et al., 2013). Figure 4 shows the individual connectivity graph for each state in the brain.

There are a few links generated only in the upregulation blocks. Two assumptions are considered about these links. They can be because of neurofeedback or recalling autobiographical paradigm. To examine these assumptions, we used a control group who received a sham neurofeedback. The activated regions of the CCMTF method in Table 1 was used as the nodes of the connectivity graph in the control group. Then, the connectivity variations from view state to upregulation state were computed for the control group. Then, the links created in the upregulation state but not in the view state were statistically compared with those in the real group and then corrected for multiple comparisons using FDR at the level of 0.05 to extract the significant links as a result of neurofeedback. These links are reported in Table 2.

**TABLE 2:** The generated links as the result of neurofeedback, obtained by the CCMTF method.

Nodes	New links
Amygdala	Caudate, inferior temporal gyrus, inferior temporal gyrus, Pallidum, The putamen, middle temporal gyrus, middle frontal gyrus, Cuneus, superior frontal gyrus, thalamus, lingual gyrus
Caudate	Putamen
Cerebellum	Inferior temporal gyrus, inferior temporal gyrus, superior parietal lobe, middle temporal gyrus, frontal operculum, lingual gyrus
Inferior temporal gyrus	Hippocampus, middle temporal gyrus
Hippocampus	Pallidum, superior frontal gyrus, superior parietal lobe, thalamus, middle temporal gyrus, Caudate, lingual gyrus
Pallidum	Putamen, middle temporal gyrus, inferior frontal gyrus,
Putamen	Superior parietal lobe, frontal operculum, Ventral Striatum, frontal operculum, middle frontal gyrus
Superior parietal lobe	Cerebellum
Thalamus	Middle temporal gyrus
Middle temporal gyrus	The occipital lobe, lingual gyrus
Middle frontal gyrus	Inferior frontal gyrus

The results of the current study are then compared with a method based on NMI.

Based on Wirsich et al. (2020), we have applied the NMI method to our datasets in three steps:

1. The EEG sources are calculated using the Minimum Norm Imaging method with BrainStorm Software (Tadel et al., 2011).
2. The connectivity between the candidate regions obtained by inverse problem and CCMTF method is calculated in each state, for EEG sources in two different frequency bands (1–15 Hz) and (15–40 Hz) and for fMRI BOLD signal. Therefore, there is a vector with a length of 50 for each corresponding connectivity between nodes.
3. The matrix of NMI is calculated between the EEG and fMRI dFCs.
4. The matrix of NMI is also calculated for EEG and fMRI dFCs for the control group.
5. The two NMI matrices are then statistically analyzed (FDR-corrected for multiple comparisons at level of 0.05).

**FIGURE 5:**
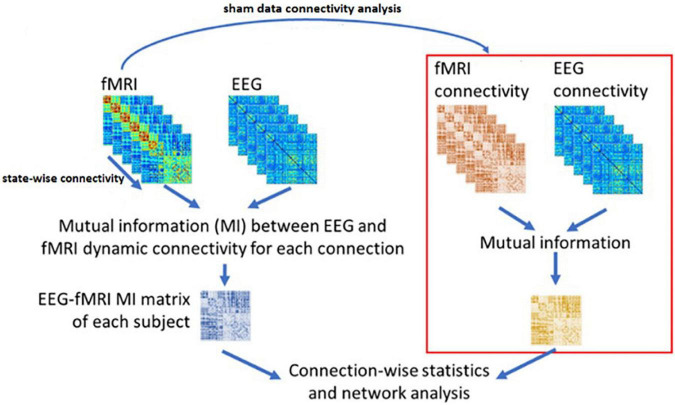
The NMI analysis for EEG and fMRI dynamic Functional Connectivity matrixes. The sham group is used to determine the statistically significant link generated as the result of neurofeedback. The figure is obtained from Wirsich et al. (2020) with a few changes.

Figure 5 illustrates the above steps for applying NMI method on the dFC of the two modalities.

**FIGURE 6:**
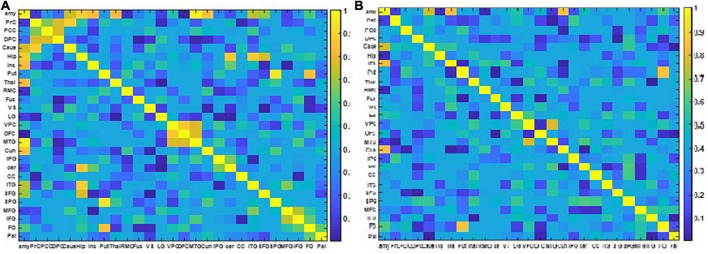
The two NMI matrixes computed from the EEG and fMRI dFC from resting state to view and upregulation states. **(A)** In the frequency range of 1–15 Hz, **(B)** in the frequency range of 15–40 Hz.

The resulting NMIs for two different EEG frequency bands are illustrated in Figure 6.

Figure 6 shows that for the frequency range of 1–15 Hz, there are more shared links between EEG and fMRI datasets and their variations across the paradigm are similar. On the other hand, for the frequency range of 15–40 Hz, a few links have similar covariation across the paradigm according to NMI criteria. The links with similar covariations between EEG and fMRI in two different frequency ranges are reported in Table 3.

**TABLE 3:** The results of the NMI method for two frequency bands.

Frequency band	Strong NMI between EEG-fMRI dFCs
1–15 Hz	Amygdala-Hippocampus, Caudate, Hippocampus, Insula, Putamen, Thalamus, Middle temporal gyrus, cuneus, Inferior temporal gyrus, Superior frontal gyrus, Middle frontal gyrus, frontal operculum Prefrontal Cingulate Cortex -Dorsolateral prefrontal cortex, Precuneus – Prefrontal Cingulate Cortex, Dorsolateral prefrontal cortex, caudate, hippocampus, Hippocampus – Cerebellum, Inferior temporal gyrus, Superior frontal gyrus, Putamen – Superior Parietal gyrus, Inferior frontal gyrus, Frontal Operculum, Cingulate Cortex Ventrolateral Prefrontal Cortex – Orbitofrontal cortex, Middle temporal cortex, Orbitofrontal cortex-middle temporal gyrus Inferior temporal gyrus – Middle frontal gyrus Inferior parietal gyrus-cerebellum Palladuim—frontal operculum
15–40 Hz	Amygdala – putamen, Cuneus, Caudate, middle temporal gyrus, frontal operculum Caudate – thalamus, Cingulate Cortex, Superior frontal gyrus Putamen-Frontal Operulum

In Table 4, the resulting links of neurofeedback are reported for each method. The results show that most of the generated links in the CCMTF method are also estimated by the NMI approach in the frequency range of 1–15 Hz.

**TABLE 4:** The extracted links as the result of neurofeedback, by CCMTF and NMI methods.

Method	Links that are extracted as the result of neurofeedback
CCMTF	Amygdala and Thalamus, caudate, middle temporal gyrus, inferior temporal gyrus, Cuneus, Middle temporal gyrus, Hippocampus and Superior frontal gyrus, inferior temporal gyrus middle temporal gyrus, occipital lobe, lingual gyrus inferior parietal lobe and cerebellum Putamen and superior parietal lobe, frontal operculum, middle frontal gyrus
NMI	Amygdala and Hippocampus, Thalamus, caudate, middle temporal gyrus, Cuneus, Middle temporal gyrus, Putamen PCC-Dorsolateral prefrontal cortex Hippocampus – Cerebellum, Superior frontal gyrus, Putamen – Superior Parietal gyrus, middle frontal gyrus, superior parietal lobe and cerebellum Ventrolateral Prefrontal Cortex – Orbitofrontal cortex, Middle temporal cortex

## Discussion

The results of the CCMTF method on the emotion regulation paradigm suggest that the frequency content of the shared component of EEG and fMRI data mostly lies in the range of 1–15 Hz. These results are expected as emotion regulation can increase the happiness and calmness in the subjects (Cisler et al., 2010). The modulation profile corresponding to the shared component shows an increasing trend from rest to view and upregulation states. These results are consistent with the results in Dehghani et al. (2020a,b) as the BOLD signal increased as a result of emotion regulation neurofeedback. The activated regions of the fMRI shared components in Table 1 are consistent with the results of Dehghani et al. (2020a). There are activations in the Cerebellum, frontal operculum, palladium which have not been detected by the GLM or ICA methods and can be the result of the joint EEG-fMRI analysis in CCMTF because of additive information during the analysis from each modality.

The results of the CCMTF method are compared with those of the NMI method (Wirsich et al., 2020). It is also shown by the NMI approach that the most common information between the two modalities is in the frequency range of (1–15) Hz. However, for the range of 15–40 Hz, there are also a few links shared between the two modalities, because NMI is a non-linear metric and can identify both linear and non-linear relationships. However, CCMTF uses a linear metric based on correlation criteria. Therefore, the CCMTF method may miss some non-linear relations between the two modalities. Another reason for the estimated links in the beta frequency band by the NMI approach is this frequency range has some overlap with the shared frequency range obtained in the CCMTF method. Both methods have estimated some common links as a result of neurofeedback and there are also some differences. These differences are the results of:

1. The changes in connectivity in the CCMTF method are obtained on the shared activated regions estimated by the CCMTF method through a simultaneous fusion approach, but in NMI, the shared links are calculated after a separate analysis on each modality.
2. The NMI method uses the inverse problem on EEG data. The inverse problem is ill-posed and cannot identify all activated sources.
3. CCMTF uses a linear criterion which may miss the non-linear relations between the two modalities.

To wrap up, the CCMTF method can estimate the shared and unshared activated region in emotion neurofeedback and making a clear correspondence with EEG frequency rhythms through one single analysis. The results of this method can be used for further analysis. CCMTF method does not require solving the inverse problem to identify the shared and unshared activated regions and the activated links in the brain between the two modalities. Besides, the relation of brain activities and their connectivity with EEG frequency bands are examined by the CCMTF method with a single analysis. The extracted connectivity links by CCMTF are among limbic, frontal, temporal, and limbic and temporal, frontal, and occipital and between temporal and occipital regions and these connectivity links can be justifies according to emotion regulation models and recalling positive autobiographical memories (Kohn et al., 2014). On the other hand, CCMTF employs a linear constraint on the shared components and misses the non-linear relations between the modalities. To gain the advantages of the CCMTF method and consider the non-linear relations, the next step is to apply a non-linear constraint in the objective function of CCMTF method.

## Conclusion

In this paper, we applied CCMTF method on a self-regulation neurofeedback paradigm using simultaneous acquisition of EEG and fMRI data. The results suggest that CCMTF method is capable to extract the shared information between two neuroimaging modalities. Comparison the calculated dFC from CCMTF analysis and those of NMI method shows that CCMTF considers linear relation between shared components, but it can extract the common dFC between EEG and fMRI without the need for inverse problem-solving. Moreover, CCMTF can extract the relation of dFCs with the brain oscillation patterns in EEG frequency bands with a single analysis.

## Data availability statement

The raw data supporting the conclusions of this article will be made available by the authors, without undue reservation.

## Ethics statement

The studies involving human participants were reviewed and approved by the ethics committees of the Iran University of Medical Sciences, Tehran, Iran. The patients/participants provided their written informed consent to participate in this study.

## Author contributions

All authors listed have made a substantial, direct, and intellectual contribution to the work, and approved it for publication.
